# Small heat shock proteins are induced during multiple sclerosis lesion development in white but not grey matter

**DOI:** 10.1186/s40478-015-0267-2

**Published:** 2015-12-22

**Authors:** Laura A. N. Peferoen, Wouter H. Gerritsen, Marjolein Breur, Kimberley M. D. Ummenthum, Regina M. B. Peferoen-Baert, Paul van der Valk, Johannes M. van Noort, Sandra Amor

**Affiliations:** Department of Pathology, VU University Medical Centre, Amsterdam, The Netherlands; Delta Crystallon BV, Leiden, The Netherlands; Blizard Institute, Barts and The London School of Medicine and Dentistry, Queen Mary University of London, London, UK

**Keywords:** Small heat shock proteins, Multiple sclerosis, White matter lesions, Grey matter lesions

## Abstract

**Introduction:**

The important protective role of small heat-shock proteins (HSPs) in regulating cellular survival and migration, counteracting protein aggregation, preventing apoptosis, and regulating inflammation in the central nervous system is now well-recognized. Yet, their role in the neuroinflammatory disorder multiple sclerosis (MS) is largely undocumented. With the exception of alpha B-crystallin (HSPB5), little is known about the roles of small HSPs in disease.

**Results:**

Here, we examined the expression of four small HSPs during lesion development in MS, focussing on their cellular distribution, and regional differences between white matter (WM) and grey matter (GM). It is well known that MS lesions in these areas differ markedly in their pathology, with substantially more intense blood-brain barrier damage, leukocyte infiltration and microglial activation typifying WM but not GM lesions.

We analysed transcript levels and protein distribution profiles for HSPB1, HSPB6, HSPB8 and HSPB11 in MS lesions at different stages, comparing them with normal-appearing brain tissue from MS patients and non-neurological controls. During active stages of demyelination in WM, and especially the centre of chronic active MS lesions, we found significantly increased expression of HSPB1, HSPB6 and HSPB8, but not HSPB11. When induced, small HSPs were exclusively found in astrocytes but not in oligodendrocytes, microglia or neurons. Surprisingly, while the numbers of astrocytes displaying high expression of small HSPs were markedly increased in actively demyelinating lesions in WM, no such induction was observed in GM lesions. This difference was particularly obvious in leukocortical lesions covering both WM and GM areas.

**Conclusions:**

Since induction of small HSPs in astrocytes is apparently a secondary response to damage, their differential expression between WM and GM likely reflects differences in mediators that accompany demyelination in either WM or GM during MS. Our findings also suggest that during MS, cortical structures fail to benefit from the protective actions of small HSPs.

**Electronic supplementary material:**

The online version of this article (doi:10.1186/s40478-015-0267-2) contains supplementary material, which is available to authorized users.

## Introduction

Multiple sclerosis (MS) is a chronic demyelinating disease of the central nervous system (CNS), affecting about 2.3 million people worldwide. Disease onset is usually first observed in young adults and it is the most common cause of non-traumatic disability in this age group [1, 2]. The pathology of MS is characterized by focal areas of inflammatory myelin damage, axonal injury and axonal loss both in white matter (WM) and grey matter (GM) [3]. Actively demyelinating WM lesions that continuously emerge and regress during disease are characterised by disruption of the blood-brain barrier, marked leukocyte infiltration, and large-scale activation of microglia/macrophages. While in the GM, demyelination generally progresses with much less tissue infiltration and microglial activation [4, 5]. In addition to focal lesions of overt myelin damage, small focal clusters of activated microglia are frequently observed in the normal appearing white matter (NAWM) of MS patients, in the absence of any detectable blood-brain barrier breakdown, leukocyte recruitment or myelin damage [6, 7]. In vivo imaging studies support the notion that these so-called preactive lesions may be the first, still reversible signs of inflammatory damage that can precede actively demyelinating MS lesions by several months [8, 9].

Previously, we have documented a close association between the development of preactive MS lesions and the presence of stressed oligodendrocytes that produce large amounts of the small heat-shock protein (HSP) alpha B-crystallin (also known as HSPB5) [10]. At later stages of lesion development, high levels of HSPB5 also emerge in astrocytes [11–13]. Elevated expression of HSPB5 by oligodendrocytes during MS may have particular relevance since HSPB5 activates a regulatory innate response by microglia via Toll-like receptor (TLR) 2 and CD14, the signs of which can already be found in preactive lesions [10, 14]. In the presence of IFN-γ as an additional co-stimulus, however, HSPB5 triggers a classical state of activation in microglia and macrophages, which is characteristic of the more advanced stages of actively demyelinating lesions [15]. HSPB5 thus offers a molecular link between stressed oligodendrocytes and the development of destructive inflammation. Since other small HSP such as HSPB1 and HSPB8 have previously been reported to share TLR agonist activity with HSPB5 [16–18], the present study addressed the question whether small HSPs other than HSPB5 display similar expression profiles during lesional development in MS.

The group of small HSPs consists of eleven members, HSPB1-HSPB10 and the recently described HSPB11 [19, 20]. However, whether HSPB11 really belongs to the family is still under debate [21]. The small HSPs share many structural and functional similarities, yet they differ in tissue distribution and expression patterns. HSPB1, HSPB5, HSPB6, HSPB8 and HSPB11 have been described to be present in the central nervous system either under normal condition or after stress induction [22–25]. Since not all small HSPs show the same response to particular types of stress [13, 23, 24, 26], differentiating their expression profiles may shed light on the molecular triggers that either drive or accompany the development of MS lesions.

In this study, we therefore evaluated by quantitative polymerase chain reaction (qPCR) and immunohistochemical staining the expression at different stages of lesion development during MS of HSPB1, HSPB6, HSPB8 and HSPB11. We paid particular attention to the cellular localisation of these four small HSPs, and additionally compared their expression between WM and GM lesions. As stated above, it has been well documented that the histopathology of GM lesions emerging during MS differs significantly from WM lesions. This difference has been suggested to be due to other pathogenic mechanisms that may operate in cortical *versus* white matter demyelination [3–5, 27]. Our data reveal that HSPB1, HSPB6 and HSPB8 but not HSPB11 are induced during active stages of demyelination in WM lesions, and particularly so in the centre of chronic active MS lesions. Different from HSPB5, however, induction of the other small HSPs was exclusively found in astrocytes and not in oligodendrocytes, and it was not found in preactive lesions. Furthermore, while the numbers of astrocytes displaying high expression of small HSPs were markedly increased in actively demyelinating lesions in the WM, no such induction was seen in GM lesions. This difference was particularly striking in leukocortical lesions that comprise both regions. Given that astrocytic expression of the small HSPs studied here is an apparently secondary response to the development of actively demyelinating MS lesions, their differential expression in either WM or GM lesions likely reflects a difference in the molecular signals that accompany inflammatory demyelination in either WM or GM during MS.

## Materials and methods

### Autopsy material

Studies were performed on post-mortem human brain tissue from 18 MS cases (mean age 62, 41–87 years) and 9 age-matched non-neurological controls (mean age 67, 51–88 years). Samples were obtained according to the protocol of the Netherlands Brain Bank (coordinator Dr. I. Huitinga, Amsterdam, The Netherlands), with the approval of the VU University Medical Ethical Committee (Amsterdam, The Netherlands). Patients and controls, or their next of kin, had given informed consent for the use of their brain tissue and clinical details for research purposes. Relevant patient information is summarised in Table 1.

**Table 1:** Clinical details of MS patients and control subjects

A. Tissues used for RT-PCR analysis					
Donor	Age	Gender	PM delay (h:m)	Diagnosis	Cause of death
1	51	M	7.30	CON	Euthanasia; esophageal cancer
2	56	M	9.15	CON	Myocardial infarction
3	62	M	7.20	CON	Unknown
4	66	F	6.00	MS (N/A)	Unknown
5	61	M	9.15	MS (SP)	Euthanasia
6	50	F	7.35	MS (SP)	Euthanasia
7	66	F	9.35	MS (PP)	Euthanasia
B. Tissues used for immunohistochemical studies					
1	51	M	7.30	CON	Euthanasia; esophageal carcinoma
2	56	M	9.15	CON	Myocardial infarction
5	61	M	9.15	MS (SP)	Euthanasia
6	50	F	7.35	MS (SP)	Euthanasia
7	66	F	9.35	MS (PP)	Euthanasia
8	82	M	6.53	CON	Pleuritis carcinomatosis
9	87	M	6.32	CON	Pneumonia
10	66	M	7.45	CON	Ruptured abdominal aorta aneurysm
11	88	M	7.00	CON	End stage rectum and prostate carcinoma
12	60	F	7.30	CON	Infection e.c.i
13	51	M	7.45	CON	Suicide
14	41	F	8.25	MS (SP)	Unknown
15	43	F	10.45	MS (SP)	Subdural haematoma, pneumonia
16	56	M	8.00	MS (SP)	Pneumonia
17	78	F	11.10	MS (N/A)	Cerebrovascular accident
18	66	M	7.30	MS (PP)	Ileus
19	77	F	10.00	MS (SP)	Euthanasia
20	45	M	7.45	MS (N/A)	Pulmonary embolism
21	83	M	7.50	MS (PP)	Aspiration pneumonia
22	74	M	10.15	MS (N/A)	Cardio-respiratory insufficiency
23	54	M	8.15	MS (PP)	Euthanasia
24	53	M	10.00	MS (SP)	Euthanasia
25	56	F	8.25	MS (SP)	Pneumonia
26	54	M	10.50	MS (N/A)	Euthanasia
27	87	F	9.30	MS (N/A)	Renal insufficiency

*PM* post mortem, *M* male, *F* female, *CON* control, *MS* multiple sclerosis, *SP* secondary progressive, *PP* primary progressive, *N/A* not known

### Real-time qPCR

Snap-frozen material of WM from control subjects and NAWM from MS patients (Table 1A) was collected in Eppendorf tubes containing 0.5 mL TRIzol® (LifeTechnologies, Bleiswijk, The Netherlands) and homogenised. Total RNA was extracted using chloroform following manufacturer’s instructions (Invitrogen, Breda, The Netherlands). RNA purity and concentrations were measured using a NanoDrop 2000 spectophotometer (Thermo Fisher Scientific, Waltman, USA). According to manufacturer’s instructions 1 μg of total RNA was reverse transcribed into cDNA (Reverse Transcription System, Promega) and real-time qPCR was carried out using Sso Advanced™ universal SYBR® Green supermix (Biorad, Veenendaal The Netherlands). The primer sequences are listed in Table 2.

**Table 2:** Primer sequences

Markers	GeneID	Sequence forward	Sequence reverse	Size (bp)	Annealing temperature (°C)
HSPB1	3315	ACGGTCAAGACCAAGGATGG	AGCGTGTATTTCCGCGTGA	104	58
HSPB6	126393	TGCTAGACGTGAAGCACTTCT	ACCACCTTGACAGCAATTTCC	49	64
HSPB8	26353	CTCCTGCCACTACCCAAGC	GGCCAAGAGGCTGTCAAGT	123	64
HSPB11	51668	TGATGGCTCCGCTACTTACTT	GCAGAAACGCTATGCACAGAT	78	60
EF1α	1915	AAGCTGGAAGATGGCCCTAAA	AAGCGACCCAAAGGTGGAT	116	55–62
18S	100008588	GTAACCCGTTGAACCCCATT	CCATCCAATCGGTAGCG	150	58–65

Reactions were normalized to the reference genes 18S (data not shown) and elongation factor 1α (EF1α). Primer pair efficiency (E) was determined using LinRegPCR software [28] and relative expression calculated as ${{\mathsf{E}}^{\hbox{-} \mathsf{C}\mathsf{T}}}^{\mathsf{target}\ \mathsf{gene}}/{\mathsf{E}}^{\hbox{-} \mathsf{C}\mathsf{T}\ \mathsf{reference}\ \mathsf{gene}}$. Melting curves and gel electrophoresis were routinely performed to define the PCR-product specificity (data not shown). All experiments were performed in triplicate.

### Lesion characterisation

MS lesions were identified by MRI-guided sampling of the CNS as described previously [29]. For lesion characterisation 5 μm-thick paraffin sections were stained with antibodies directed against myelin proteolipid protein (PLP) and HLA-DR to detect activated microglia/macrophages. Slides were deparaffinised in xylene and rehydrated through descending alcohol concentrations. Endogenous peroxidase activity was blocked by incubating the slides 30 min in phosphate buffered saline (PBS) containing 0.3 % H_2_O_2_. For HLA-DR expression the sections were heated in 0.01 M citrate buffer (pH 6.0). After allowing the heated sections to regain room temperature (RT), sections were rinsed in PBS. Sections were incubated with primary antibodies directed against PLP and HLA-DR (Table 3) for 1 h at RT. After incubation, sections were thoroughly rinsed in PBS and incubated with ready-to-use goat-anti-mouse EnVision^+^™-HRP (Dako, Glostrup, Denmark) for 30 min at RT. Sections were rinsed in PBS, incubated in 3,3’-diaminobenzidine (DAB; Dako) to visualise staining and counterstained with haematoxylin.

**Table 3:** Antibodies used for immunohistochemistry

Antigen	Clone	Species	Isotype	Dilution	Antigen retrieval	Manufacturer
PLP	Plpc1	Mouse	IgG2a	1:3000	none	AbD Serotec
HLA-DR	LN3	Mouse	IgG2b	1:1000	Citrate buffer	eBioscience
Olig2	Polyclonal	Rabbit		1:200	Citrate buffer	Millipore
GFAP	6 F2	Mouse	IgG1	1:200	Citrate buffer	Monosan
HSPB1	EPR7278	Rabbit	IgG	1:500	Citrate buffer	Abcam
HSPB5	JAM01	Mouse	IgG1	1:100	Citrate buffer	In house
HSPB6	EPR14458	Rabbit	IgG	1:500	Citrate buffer	Abcam
HSPB8	Polyclonal	Rabbit		1:500	Citrate buffer	Abcam
HSPB11	polyclonal	Rabbit		1:50	Citrate buffer (20 min)	Novus

*PLP* proteolipid protein, *HLA-DR* human leukocyte antigen, *GFAP* glial fibrillary acid protein, *HSPB* heat shock protein B

White matter MS lesions were classified according to the degree of myelin damage and the activity of the microglia/macrophages as described earlier [6]. Briefly, preactive lesions were classified as a cluster of activated (HLA-DR^+^) microglia in NAWM; active lesions were characterised by a focal area of myelin loss filled with myelin-laden ‘foamy’ macrophages; chronic active lesions were identified by a rim of activated microglia/macrophages surrounding a hypocellular centre, and inactive WM lesions as a demyelinated area with few or no HLA-DR^+^ cells.

In contrast to WM, cortical demyelination during MS is not accompanied by fulminant activation of microglia/macrophages. Therefore, cortical lesions were classified according to the neuroanatomical location as reported previously [27, 30]. Lesions of myelin damage in the white matter that extend beyond the border between white and grey matter were classified as leukocortical lesions, whereas lesions completely within the cortex are termed intracortical, and demyelination along the surface of the brain as subpial. For this study 23 paraffin-embedded tissue blocks containing 15 preactive, 8 active, 6 chronic active and 4 inactive WM lesions, and an additional 5 leukocortical, 3 intracortical and 7 subpial GM lesions were examined.

### Immunohistochemical detection of HSPB1, HSPB6, HSPB8, HSPB11

To analyse the expression of small HSPs, paraffin sections were double labelled for HLA-DR and small HSPs. Deparaffinised, rehydrated and endogenous peroxidase-blocked slides were heated in a microwave oven for 10 min in 0.01 M citrate buffer (pH 6.0). After antigen retrieval, sections were allowed to cool to RT and rinsed in PBS. Subsequently, sections were incubated overnight at RT with primary antibodies directed to HLA-DR and HSPB1, HSPB6, HSPB8 or HSPB11 (Table 3). After the sections were rinsed, the relevant secondary antibodies [goat-anti-mouse-alkaline phosphatase (AF labelled (Southern Biotech, AL, USA) or goat-anti-rabbit EnVision^+^™ -HRP (Dako)] were applied for 1 h at RT. Cells labelled with HRP were visualised with DAB, whereupon slides were rinsed in Tris buffered saline (TBS) to detect AF-labelled cells with liquid permanent red substrate-chromogen (Dako). The sections were counterstained with haematoxylin prior to permanent mounting in Aquatex (Merck, Darmstadt, Germany). Negative controls were performed by eliminating the primary antibodies and by replacing the primary antibodies by appropriate isotype controls, *viz.* mouse IgG2b (BD Bioscience, Breda, the Netherlands) or rabbit IgG (Abcam, Cambridge, UK).

### Quantitative analysis

Lesions were selected based on the integrity of myelin as judged by PLP expression and the presence of HLA-DR^+^ cells. Areas within the lesion were selected randomly and pictures of the same areas were taken in sequential sections double stained for the selected small HSPs and HLA-DR. Pictures were taken with a Leica DC500 (Leica Microsystems, Heidelberg, Germany) at 200× magnification. ImageJ software [31] was used to analyse the picture, numbers of nuclei and positive cells were counted manually using the cell-counter plugin (de Vos, University of Sheffield, UK). Taking into account that, as a result of differences in levels of infiltration, the absolute numbers of cells per area differ between white and grey matter as well as between the distinct lesion types, immune positive cells are presented as relative numbers to total number of nuclei. All nuclei visible on the pictures were counted, with the exception of those contained within blood vessels. To verify accuracy of the data, 18 pictures were counted by two independent observers and inter-observer consistency was evaluated, resulting in a correlation coefficient of 0.99.

### Double immunolabelling

To identify the cells expressing the selected small HSPs, double labelling with antibodies directed to glial fibrillary acidic protein (GFAP), an astrocytic marker, or Olig2, an oligodendroglial marker, was performed. Double labelling of paraffin wax sections was carried out sequentially as follows. Deparaffinised sections were subjected to antigen retrieval and incubated overnight with primary antibodies directed to GFAP or Olig2 (Table 3). After thorough rinsing, goat-anti-mouse Alexa594-labelled (Life technologies, OR, USA) and goat-anti-rabbit-EnVision^+^™ -HRP-labelled secondary antibodies for GFAP and Olig2 respectively were incubated at RT for 1 h. DAB chromogen was used to visualise HRP-labelled oligodendrocytes. Given that antibodies directed to both Olig2 and the small HSPs are produced in rabbits, an additional antigen retrieval step after the DAB visualisation was performed. After which, sections were incubated with antibodies directed against HSPB1, HSPB5, HSPB6, HSPB8 or HSPB11 at RT for 24 h. After washing, the secondary antibodies goat-anti-rabbit Alexa-488 (Life technologies, OR, USA) for GFAP and goat-anti-rabbit-AF (Southern Biotech, AL, USA) for Olig2 double labelling were allowed to bind for 1 h at RT. Liquid permanent red (LPR) substrate was used to detect AF-labelled cells. Sections were counterstained with haematoxylin for the immunohistochemical procedure or with 0.2 μg/mL 4’,6-diamidino-2-phenylindole (DAPI; Invitrogen) in PBS for the immunofluorescence protocol. To examine whether cells expressed more than one small HSP, sections were also double labelled for combinations of two small HSPs. Immunohistochemistry was performed as explained above for double staining with Olig2 using the relevant antibodies for HSPB1, HSPB5 and HSPB8 to interact with HRP-labelled secondary antibodies and visualise with DAB. Thereafter slides were heated in citrate buffer (pH 6.0) at 90 °C and antibodies for HSPB1, HSPB5 or HSPB6 were used to interact with AP-labelled antibodies and visualised with LPR. Immunofluorescence pictures were taken using a Leica DM 5500 supported by LAS-AF 3.0.0 software, immunohistochemical photographs were taken with a Leica DC500 microscope.

### Statistical analysis

Statistical analysis was performed using GraphPad Prism software 6.0 (GraphPad Software, San Diego, CA). Data distribution was tested for normality with the D’Agostino-Pearson normality test. Since the data were not normally distributed, data were analysed using non-parametric tests. Counts from control white and grey matter were compared to NAWM and NAGM from MS patients, respectively, using the Mann-Whitney U test. Differences between MS lesion types were analysed using the Kruskal-Wallis Test. When positive, Mann-Whitney U test was performed to test subgroups. Differences were considered to be significant when *p* < 0.05.

## Results

### Expression of small HSPs is already enhanced in normal-appearing white matter during MS

**Fig. 1:**
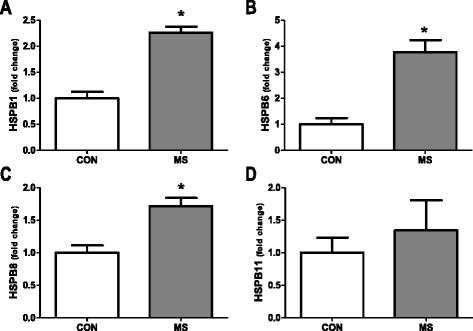
Transcript levels of small heat shock proteins in control white matter and NAWM in MS patients. Real time PCR analysis of transcripts encoding HSPB1 (**a**), HSPB6 (**b**), HSPB8 (**c**) and HSPB11 (**d**) comparing white matter from non-neurological controls (*n* = 3) and MS patients (*n* = 4). Data represent mean ± SEM and are expressed relative to the housekeeping gene EF-1α. *p < 0.05

Transcript levels of HSPB1, HSPB6, HSPB8 and HSPB11 were determined by qPCR in WM tissue samples from non-neurological controls and NAWM from MS patients. Transcript levels of HSPB1, HSPB6 and HSPB8 were significantly upregulated in NAWM tissue from MS patients as compared to controls, with fold inductions of 2.3, 3.8 and 1.7 respectively (*p* < 0.05; Fig. 1a–c). In contrast, HSPB11 mRNA levels did not show significant differences between the two groups (Fig. 1d). These data are in line with several reports documenting ongoing diffuse inflammation and other abnormalities in the NAWM of MS patients [32], including increased levels of another member of the family of small HSPs, *viz*. HSPB5 (Additional file 1: Figure S1) [33].

### Expression of HSPB1 in white matter MS lesions

To examine in more detail the expression pattern of HSPB1 in WM lesions from MS patients, we first performed double staining for PLP and HLA-DR to evaluate the extent of demyelination and microglial/macrophage activation, respectively. Double immunolabelling was thereafter performed using antibodies directed against HSPB1 and HLA-DR. This approach enabled the identification of the different lesion types. A total of 15 preactive, 8 active, 6 chronic active and 4 inactive lesions were compared to 5 areas of NAWM. In addition, 7 tissue blocks from non-neurological controls were analysed.

**Fig. 2:**
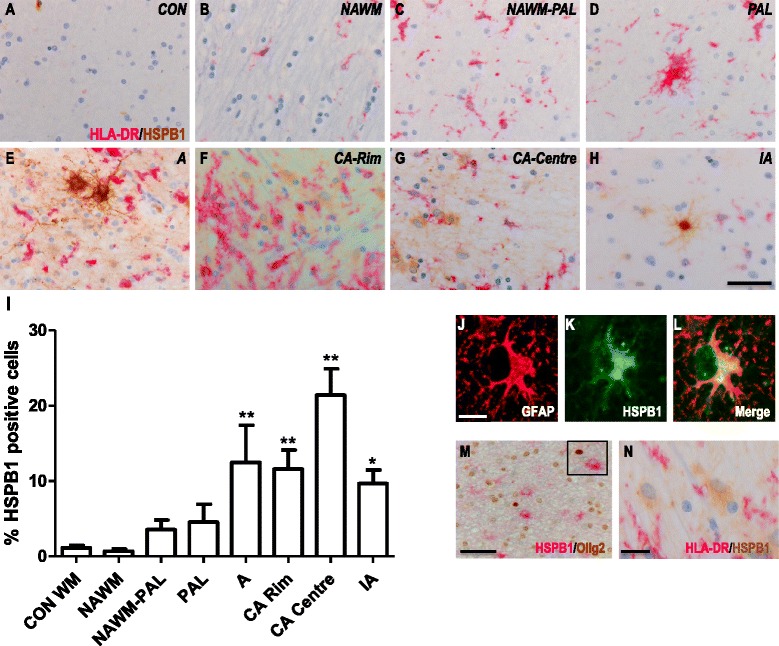
HSPB1 expression in white matter MS lesions. Representative images of white matter tissues from non-neurological controls (**a**) and MS cases (**b**–**h**) double labelled for HLA-DR (pink) and HSPB1 (brown). NAWM (**b**), NAWM surrounding a preactive lesion (**c**), preactive lesion (**d**), active lesion (**e**), chronic active lesion (**f**- rim, **g** - centre) and inactive lesions (**h**) from MS cases. Quantification of HSPB1^+^ cells in lesion areas (**i**). GFAP^+^ astrocytes co-localise with HSPB1 (**j**–**l**), while Olig2^+^ oligodendrocytes (**m**) or HLA-DR^+^ microglia (**m**) do not express HSPB1. Percentages of cells in each area (**i**). Data are shown as mean ± SEM. Significance was analysed between the control and NAWM, or the NAWM group and the different lesion types using the Mann-Whitney U test. *p < 0.05, **p < 0.01. Scale bar (**a**–**h**), (**m**–**n)** = 50 μm, (**j**–**l**) = 10 μm. CON = control, NAWM = normal appearing white matter, PAL = preactive lesions, A = active lesion, CA = chronic active lesion, IA = inactive lesions

Marginal expression of HSPB1 was detected in the parenchyma of control WM, with only 1 % of the nuclei showing a cytoplasmic staining pattern (Fig. 2a, i). However, intense expression was detected in the endothelial and smooth muscle layers of both smaller and larger blood vessels in controls (data not shown). A similar pattern was observed in the NAWM of MS patients, where HSPB1 was expressed in blood vessels but scarcely in the parenchyma (Fig. 2b, i). In actively demyelinating lesions, prominent HSPB1 immunoreactivity was exclusively associated with astrocytes as demonstrated by GFAP co-localization (Fig. 2j–l), and supported by the astrocytic morphology of HSPB1^+^ cells (Fig. 2e–h). In contrast, HSPB1 expression was not associated with oligodendrocytes or microglia, as evidenced by the lack of any co-localization between HSPB1 and either Olig2 or HLA-DR, respectively (Fig. 2m, n).

Quantitating the frequency HSPB1^+^ astrocytes revealed a significant increase in the numbers of such cells in active lesions, comparable with the increase observed at the rim of chronic active lesions (12.5 % positive cells; Fig. 2i). The highest frequency of HSPB1^+^ astrocytes was found in the hypocellular centre of chronic active lesions, in which as many as 25 % of all astrocytes were positive for HSPB1. In inactive MS lesions, the frequency of HSPB1^+^ astrocytes was much lower again, although still significantly higher as compared to NAWM (*p* < 0.05; Fig. 2i). Sections stained with isotype control antibodies (rabbit IgG and mouse IgG2b) or treated while omitting the primary antibodies were consistently negative for both MS patients and controls (data not shown).

### Expression of HSPB6 in white matter MS lesions

**Fig. 3:**
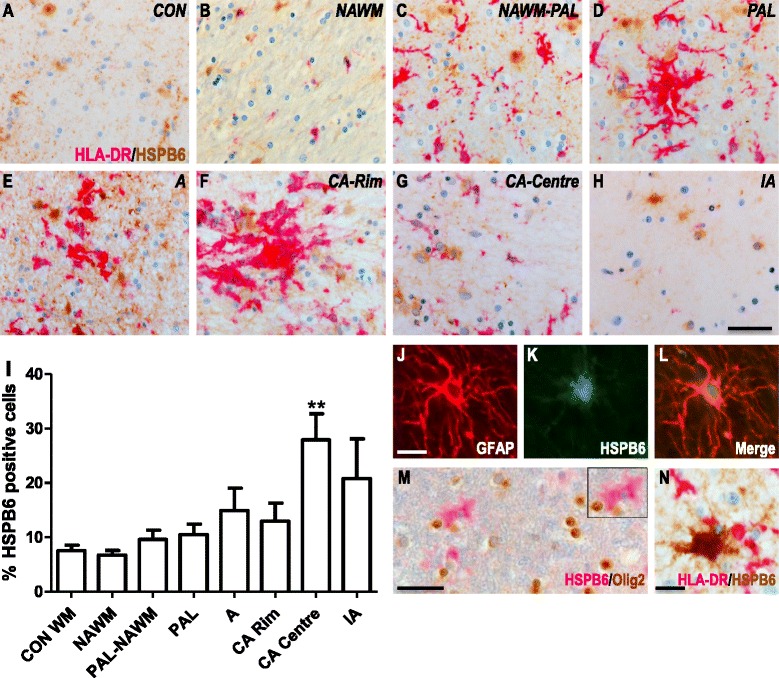
HSPB6 expression in white matter MS lesions. Representative images of white matter tissues from non-neurological controls (**a**) and MS cases (**b**–**h**) double labelled for HLA-DR (pink) and HSPB6 (brown). NAWM (**b**), NAWM surrounding a preactive lesion (**c**), preactive lesion (**d**), active lesion (**e**), chronic active lesion (**f**- rim, **g** - centre) and inactive lesions (**h**) from MS cases. Quantification of HSPB6+ cells in lesion areas (**i**). GFAP+ astrocytes co-localise with HSPB6 (**j**–**l**), while Olig2+ oligodendrocytes (**m**) or HLA-DR+ microglia (**n**) do not express HSPB6. Percentages of cells in each area (**I**). Data are shown as mean ± SEM. Mann-Whitney U tests were performed for comparisons between control white matter and NAWM and between NAWM and the different lesion types. **p < 0.01. Scale bar (**a**–**h**, **m**) = 50 μm, (**j**–**l**, **n**) = 10 μm. CON = control, NAWM = normal appearing white matter, PAL = preactive lesions, A = active lesion, CA = chronic active lesion, IA = inactive lesions

Similar to HSPB1, expression of HSPB6 was detected in blood vessels in control tissue samples where the smooth muscle cells of large vessels were positive, while smaller vessels and endothelial cells were negative for HSPB6. Some HSPB6 immunolabelling was also observed in the parenchyma of non-neurological controls (Fig. 3a) but evaluation of the frequency of astrocytes displaying high expression of HSPB6 revealed no significant differences between control WM (7.5 ± 2.7 %) and NAWM in MS patients (6.7 ± 1.9 %) (Fig. 3a, b, i). Evaluation of MS lesions again revealed exclusive induction of HSPB6 in astrocytes, as confirmed by co-localization of HSPB6 with GFAP (Fig. 3j–l) but not with Olig2 (Fig. 3m) or HLA-DR (Fig. 3n), and supported by the morphology of HSPB6^+^ cells. Although a slight increase in numbers of HSPB6^+^ astrocytes was observed in and around both active and inactive lesions, the frequency of HSPB6^+^ astrocytes was significantly increased relative to control WM only in the centre of chronic active lesions (Fig. 3g, i, *p* < 0.01).

### HSPB8 expression in white matter MS lesions

**Fig. 4:**
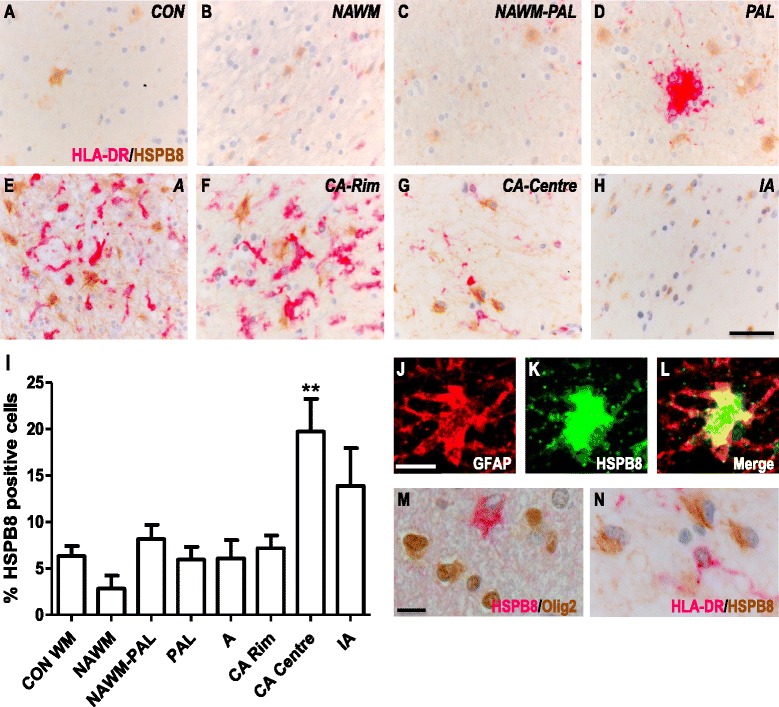
HSPB8 expression in white matter MS lesions. Representative images of white matter tissues from non-neurological controls (**a**) and MS cases (**b**–**h**) double labelled for HLA-DR (pink) and HSPB8 (brown). NAWM (**b**), NAWM surrounding a preactive lesion (**c**), preactive lesion (**d**), active lesion (**e**), chronic active lesion (**f**- rim, **g** - centre) and inactive lesions (**h**) from MS cases. Quantification of HSPB8+ cells in lesion areas (**i**). GFAP+ astrocytes co-localise with HSPB8 (**j**–**l**), while Olig2+ oligodendrocytes (**m**) or HLA-DR+ microglia (**n**) do not express HSPB8. Percentages of cells in each area (**I**). Data are shown as mean ± SEM. Mann-Whitney U tests were performed for comparisons between control white matter and NAWM and between NAWM and the different lesion types. **p < 0.01. Scale bar (**a**–**h**) = 50 μm, (**j**–**n**) = 10 μm. CON = control, NAWM = normal appearing white matter, PAL = preactive lesions, A = active lesion, CA = chronic active lesion, IA = inactive lesions

Similar to HSPB1 and HSPB6, expression of HSPB8 was restricted to GFAP^+^ astrocytes as determined by double labelling for GFAP, and absent from Olig2^+^ oligodendrocytes or HLA-DR^+^ microglia (Fig. 4). In contrast to HSPB1 and HSPB6, however, no vascular expression of HSPB8 was observed (data not shown). The frequencies of HSPB8^+^ astrocytes were very similar in control tissues (6.3 ± 2.7 %, Fig. 4i), NAWM (2.8 ± 3.2 %), NAWM close to preactive lesions (5.9 ± 5.3 %), preactive lesions (8.15 ± 5.9 %), active lesions (6.1 ± 5.5 %), and the rim of chronic active lesions (7.17 ± 3.4 %) (Fig. 4a–i). Like in the case of HSPB6, a significantly increased frequency of HSPB8^+^ astrocytes was only found in the centre of chronic active lesions (19.7 ± 8.6 %, *p* < 0.01). In inactive lesions the number of HSPB8^+^ cells was not significantly different from NAWM (13.8 ± 8.1 %).

### Expression of HSPB11 in white matter MS lesions

**Fig. 5:**
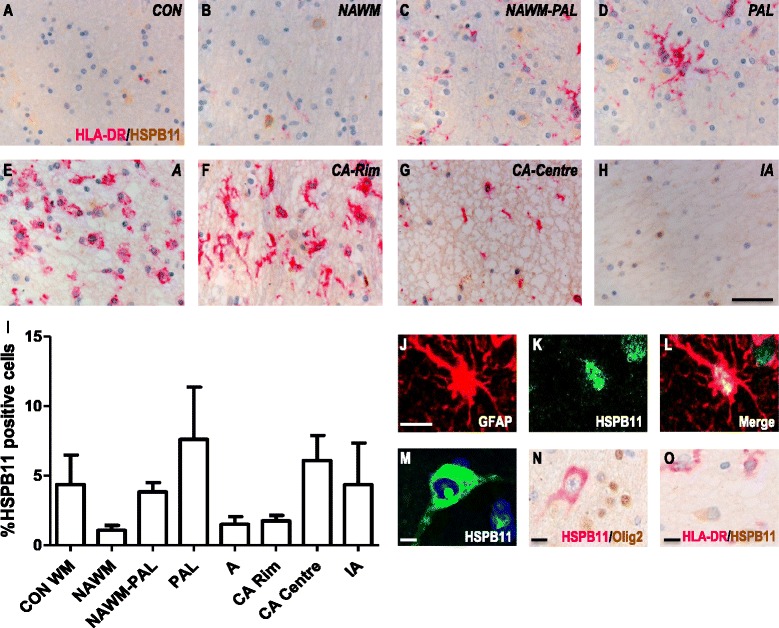
HSPB11 expression in white matter MS lesions. Images of white matter tissues from non-neurological controls (**a**) and MS cases (**b**–**h**) double labelled for HLA-DR (pink) and HSPB11 (brown). NAWM (**b**), NAWM surrounding a preactive lesion (**c**), preactive lesion (**d**), active lesion (**e**), chronic active lesion (**f**- rim, **g** - centre) and inactive lesions (**h**) from MS cases. Quantification of HSPB11+ cells in lesion areas (**i**). HSPB11 co-localises with GFAP+ astrocytes (**j**–**l**), and is expressed by white matter neurons (**m**) while Olig2+ oligodendrocytes (**n**) or HLA-DR+ microglia (**o**) do not express HSPB11. Percentages of cells in each area (**i**). Data are shown as mean ± SEM. Statistical analysis is performed by Mann- Whitney U test, comparing control white matter and NAWM or NAWM and the separate lesion types, no significant differences are found. Scale bar (**a**–**h**) = 50 μm, (**j**–**o**) = 10 μm. CON = control, NAWM = normal appearing white matter, PAL = preactive lesions, A = active lesion, CA = chronic active lesions, IA = inactive lesions

While some HSPB11 immunoreactivity was detected, staining intensity was weak in control WM (Fig. 5a) as well as in the various MS samples. Additionally, no marked changes in areas of demyelinating lesions were observed (Fig. 5b–h, i). In agreement with the qPCR data (Fig. 1d), therefore, the overall expression pattern of HSPB11 did not differ between control cases and MS patients. When detectable, HSPB11 was exclusively found in astrocytes (Fig. 5j–l) but not in oligodendrocytes (Fig. 5n) or microglia (Fig. 5o). Occasionally, HSPB11^+^ cells appeared to resemble WM neurons (Fig. 5m).

### Expression of small HSPs in grey matter MS lesions

**Fig. 6:**
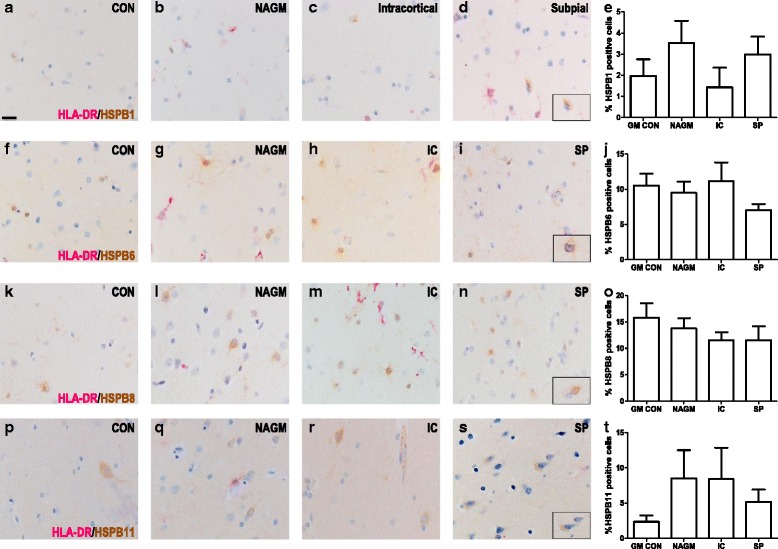
Expression pattern of the small HSP in cortical MS lesions. Grey matter from non-neurological cases and NAGM, intracortical demyelination and subpial lesions from MS cases were double labelled with HLA-DR (*pink*) and the small HSPs (*brown*), HSPB1 (**a**), HSPB6 (**b**), HSPB8 (**c**) or HSPB11 (**d**). Quantification revealed no correlation between the expression pattern of the small HSPs and cortical damage (A5, B5, C5, D5). NAGM was compared versus the control, intracortical and subpial groups by the use of Mann-Whitney U test. Scale bar = 25 μm. CON = control, NAGM = normal appearing grey matter, IC = intracortical, SP = subpial

Demyelinating lesions in the GM differ substantially from WM lesions with regard to the degree of visible inflammation as reflected by blood-brain barrier damage, leukocyte infiltration and microglial activation [4]. To examine whether such differences impact on the expression of small HSPs, we examined their expression in 3 intracortical lesions (from 3 different patients) and 7 subpial lesions (from 6 different patients) and compared the results with those found for normal-appearing grey matter (NAGM, *n* = 4) and GM samples collected from non-neurological controls (*n* = 4; Fig. 6).

Similar to control WM, HSPB1 expression in control GM was detected in the endothelium and smooth muscle cells of blood vessels and leptomeningeal vessels (data not shown). HSPB1^+^ cells, identified as astrocytes by double staining for GFAP, were rarely observed in the GM parenchyma in controls (Fig. 6a, e; 1.97 ± 1.59 %). In the NAGM of MS patients, and even in demyelinating cortical lesions, very similar low frequencies were found for HSPB1^+^ astrocytes (Fig. 6a–e). Also when examining expression of the other small HSPs in MS patients we found no significant differences between demyelinating GM lesions and NAGM (Fig. 6b–d). When detectable in the GM, the expression of small HSPs was exclusively confined to astrocytes again. Different from its expression profile in WM, HSPB6 immunolabelling also revealed some nuclear staining in GM astrocytes. Some cells expressing HSPB11 morphologically resembled neurons, which were otherwise negative for HSPB1, HSPB6 and HSPB8.

### The expression of small HSPs is different between the white and grey matter areas of leukocortical lesions

Leukocortical lesions affect both the deeper layers of the GM and the adjacent WM. The GM parts are more inflammatory in comparison to subpial and intracortical lesions, yet the number of activated HLA-DR^+^ microglia is approximately 5 times lower than in the corresponding WM part of the lesion [34, 35]. To analyse whether this difference is associated with an altered expression of small HSPs, we evaluated leukocortical lesions from 5 MS patients.

**Fig. 7:**
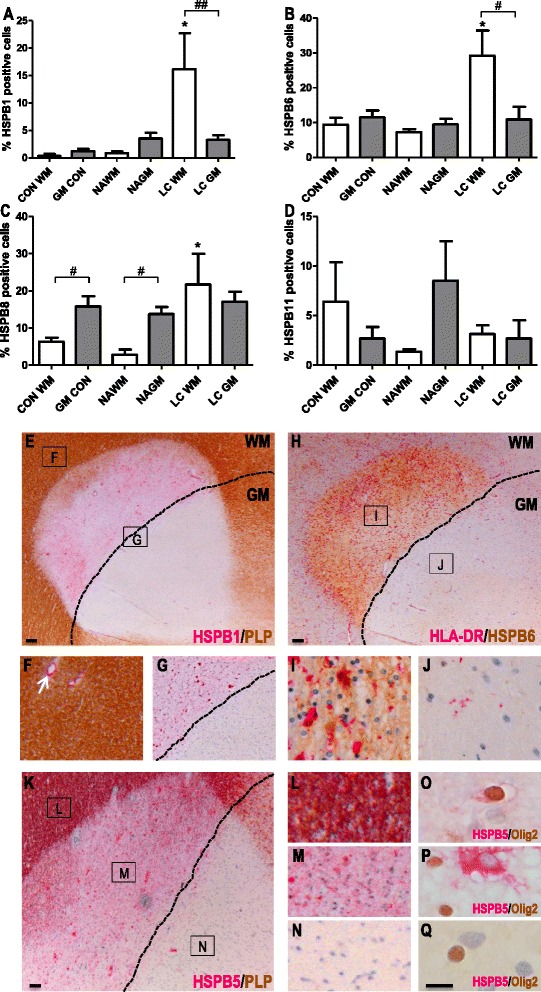
Small HSP expression in leukocortical MS lesions. Expression of HSPB1 (**a**), HSPB6 (**b**), HSPB8 (**c**) and HSPB11 (**d**) was quantified in the white matter (*white bars*) and grey matter (*grey bars*) area of leukocortical MS lesions compared to NAWM and NAGM in MS cases, and control white and grey matter in controls. **e** A leukocortical lesion identified by a focal area of myelin loss depicted by PLP staining (*brown*). The border between WM and GM is depicted by a black line (also in **g**, **h** and **k**). Immunohistochemistry shows the marked difference in HSPB1 expression in the WM and GM parts of the LC lesion. **f** In the NAWM HSPB1 expression is limited to blood vessels (arrow). **g** Enlargement of area in (**e**) shows HSPB1 expression (*pink*) is restricted to the white matter part of the lesion. **h** HSPB6 expression (brown) in an active leukocortical lesion containing HLA-DR+ microglia/macrophages (pink) restricted to the WM part (**i**). HSPB6 is highly expressed in the WM part (**i**) and absent in the grey matter part (**j**). **k** A leukocortical lesion identified by a focal area of myelin loss depicted by PLP staining (brown). HSPB5 (pink) is selectively expressed in oligodendrocytes in preactive lesions (data not shown) in NAWM close to the WM/GM border (**l**, **o**), while in the active WM part (**m**) HSPB5 accumulates also in astrocytes (**p**) but not in the GM part of the lesion (**n**, **q**). Statistical analysis (**a**–**d**) is performed by a Mann-Whitney U test, comparing white and grey matter groups with each other (#) or comparing NAWM or NAGM with the control or lesion group (*) . # *p* < 0.05; ## *p* < 0.01; *p < 0.05. Scale bars **e**, **h**, **k** = 100 μm; **o**–**q** = 50 μm; **f**–**g**, **i**–**j**, **l**–**n** = digitally zoomed in. CON = control, WM = white matter, GM = grey matter; NAWM = normal appearing white matter, NAGM = normal appearing grey matter, LC = leukocortical lesions

While very similar numbers of HSPB1^+^ astrocytes were found in control WM, control GM, NAWM and NAGM (Fig. 7a), the number of HSPB1^+^ astrocytes significantly increased in the WM part of the lesion as compared to control WM and NAWM. Strikingly, however, the numbers of HSPB1^+^ astrocytes in the GM part of the lesion were about five times lower than those found in the WM parts of the very same lesions (*p* < 0.01, Fig. 7a). In addition, extracellular structures in the WM part of the lesion but not the GM part were clearly stained for HSPB1 as well (Fig. 7e–g). A very similar result was obtained when evaluating the expression of HSPB6. Again, HSPB6 expression in the WM part of the lesion was significantly (*p* < 0.05) higher as compared to the GM part (Fig. 7b, h–j). While evaluating HSPB8 staining, we somewhat surprisingly found that already in control GM samples or unaffected GM regions from MS patients, the frequency of HSPB8^+^ astrocytes was higher than in WM regions. Still, and similar to the other two small HSPs, the numbers of HSPB8^+^ astrocytes were strongly increased in the WM but not the GM part of leukocortical lesions (Fig. 7c). Evaluation of the expression profile of HSPB11 demonstrated that the very low levels of expression of HSPB11 did not significantly change in either part of leukocortical lesions (Fig. 7d), in line with the other data for HSPB11 as described above.

Prompted by the striking differences in expression of HSPB1, HSPB6 and HSPB8 in the WM *versus* GM part of leukocortical MS lesions, we also examined HSPB5 for this particular feature. Similar to HSPB1 and HSPB6, high expression levels of HSPB5 were restricted to the WM part of the lesions (Fig. 7k, m). In line with previous data, HSPB5 expression in the WM part was found not only in astrocytes, but also in oligodendrocytes (Fig. 7o, p, q).

### Subsets of astrocytes in MS lesions co-express small HSPs

**Fig. 8:**
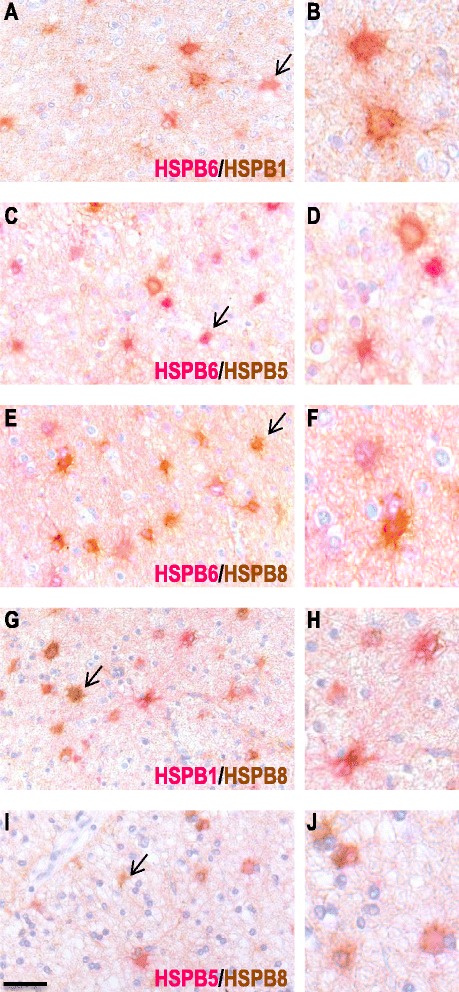
Double immunolabelling for combinations of different sHSPs. Representative images of active white matter lesions double labelled for HSPB6 (*pink*) and HSPB1 (*brown*; **a**–**b**), HSPB6 (*pink*) and HSPB5 (*brown*; **c**–**d**), HSPB6 (*pink*) and HSPB8 (*brown*; **e**–**f**), HSPB1 (*pink*) and HSPB8 (*brown*; **g**–**h**) and HSPB5 (*pink*) and HSPB8 (*brown*; **i**–**j**). Most astrocytes are double labelled, yet some show single staining; examples are indicated by the arrows. Scale bar = 50 μm, **b**, **d**, **f**, **h**, **j** are digitally zoomed in

To examine whether HSPB1, B5, B6 and B8 were induced in the same subset of astrocytes we performed double staining for combinations of HSPBs. These studies revealed that while HSPB6 is already expressed in the NAWM, subsets of astrocytes co-express with HSPB1 and HSPB5 in active demyelinating lesions. However, some astrocytes were more intensely stained with either only HSPB1 or HSPB5 (Fig. 8a–d). In contrast, all astrocytes expressing HSPB8 co-localised with HSPB1, HSPB5 and HSPB6 in active MS lesions, yet a subset of astrocytes only expressed HSPB8 (Fig. 8g–j). In the NAWM a small percentage of astrocytes exclusively stained for HSPB8, all astrocytes positive for HSPB6 were double labelled (data not shown).

## Discussion

Despite their generally recognized relevance to cellular survival, cellular migration and to tissue remodelling and repair [36, 37], surprisingly little is known about the role of small HSPs in MS. With the exception of HSPB5, only limited data are available on their expression profiles during the development of MS lesions, even though such profiles may hold important clues to molecular factors involved in the disease process. In the present study, we examined the expression of four small HSPs during MS, and found significant increases in transcript levels for HSPB1, HSPB6 and HSPB8 but not for HSPB11 in NAWM from MS patients compared to white matter from non-neurological controls. Immunohistochemical studies revealed increased expression of HSPB1, HSPB6 and HSPB8 in actively demyelinating white matter MS lesions, exclusively found in astrocytes.

Especially since the most prominent expression was observed in the centre of chronic active lesions, astrocytic expression of these small HSPs is most likely a secondary response to lesion development. This sets these small HSPs apart from another family member, *viz.* HSPB5 which is found in MS lesions at increased levels not only in astrocytes but also in oligodendrocytes [10–12, 14, 15] (Additional file 1: Figure S1), and selectively accumulates in oligodendrocytes already at the stage of preactive lesions in the absence of infiltration or myelin damage. The most striking finding of the present study, however, is that the induction of small HSPs in astrocytes during lesion development in MS is restricted to WM and absent from GM. This difference became particularly obvious by examining the expression of small HSPs in leukocortical lesions that extend over both regions. This finding strongly suggests that the molecular signals that accompany inflammatory demyelination in either region are different, inducing marked accumulation of small HSPs in astrocytes in the WM but not in the GM.

Small HSPs are important for cell migration, differentiation, and the regulation of autophagy and apoptosis during embryonic development and tissue remodelling in the brain. During MS, the prominent expression of HSPB1, 5, 6 and 8 in astrocytes especially in chronic active lesions is therefore likely a reflection of astrocytic participation in tissue remodelling triggered by lesion formation. An involvement of HSPB1 in tissue remodelling and repair is supported for example by the observation that HSPB1-deficient mice display impaired wound healing following excisional cutaneous wounds [38]. Also in line with this notion are the many reports of increased levels of small HSP in reactive astrocytes following acute neurotrauma [39–42] and during chronic neurodegenerative disease [43, 44]. Together with our own findings, these data point to small HSP expression in astrocytes being a secondary response to damage, and playing a role in controlling inflammation and promoting tissue repair. It is of interest to note that in their presently documented expression profiles, HSPB1, 6 and 8 markedly differ from HSPB5. While HSPB5 follows the same pattern of expression in astrocytes during lesion development in MS, it differs in showing high expression in oligodendrocytes as well, and doing so already in preactive MS lesions, even before leukocyte infiltration and myelin damage develops. Such differences may help shed some light on the molecular factors that operate at different stages of lesion development since in vitro studies indicate that small HSPs respond in different ways to certain stressors. While oxidative stress for example is an effective inducer of HSPB5 in cultured rat oligodendrocytes, it fails to induce HSPB1 [26, 45]. Similar differential responses by small HSPs have been reported for astrocytes [13, 31]. In active demyelinating lesions the majority of astrocytes do express more than one small HSP. However variation between subsets could be observed. Pathological studies are only a snapshot in time, in vitro studies have documented differences in the time course of upregulation of the various small HSPs [13, 23, 26, 45].

Our data on HSPB11 are different from the other small HSPs in that HSPB11 was not induced during the development of MS lesions. Also different from the other small HSPs, we occasionally found somewhat higher levels of HSPB11 in neurons. While HSPB11 is apparently stress inducible in some cells and shares structural homology to the other members of the family of small HSPs, the classification of HSPB11 as a *bona fide* small HSP is still under debate [20, 21]. Similar to other family members, HSPB11 has been reported to inhibit apoptosis and reduce the neurotoxicity of amyloid aggregates [46, 47] but marked differences in the neuronal expression profile of HSPB11 as compared to other small HSPs have been reported before [26]. Our somewhat deviant results for HSPB11 are therefore in line with previous reports.

Our finding that HSPB1, HSPB5, HSPB6 and HSPB8 all fail to be induced in MS lesions in GM regions, including the GM part of leukocortical lesions, extends previous reports of striking pathological differences between WM and GM demyelination in MS [3, 4, 34]. The clear signs of blood-brain barrier disruption, leukocyte influx and microglial activation that characterise actively demyelinating WM lesions are absent from GM lesions, indicating that important differences exist in the molecular make up between these lesions. Differences in the local cell populations may help explain this. An obvious difference is the presence of large numbers of oligodendrocytes and large amounts of myelin in WM, and of neurons in GM, either of which may impact on the course of inflammation. Oligodendrocytes for example produce HSPB5 which impacts on the activation state of microglia [15, 48], and myelin ingestion by microglia/macrophages has similar regulatory effects [49, 50]. Also neurons may influence local inflammatory processes since they secrete a range of mediators that regulate microglial activity, such as fraktalkine, IL-34 and extracellular nucleotides [3, 51–53]. Not only signalling mediators released during inflammation may therefore be different between WM and GM lesions, differences may also exist at the receiving end since protoplasmic astrocytes in the white matter have different origins from the fibrous astrocytes that are found in GM [54]. This may additionally cause differential astrocyte expression profiles of small HSPs during inflammation, similar to previously reported differences in the expression of S100 protein [55, 56].

## Conclusion

Our data clarify that several small HSPs including HSPB1, HSPB6 and HSPB8 are selectively induced in astrocytes as a secondary response to the development of MS lesions in WM but not GM areas of the CNS. They thus extend several previous reports on marked pathological differences between WM and GM lesions in MS. Since the induction of these small HSPs in astrocytes is clearly secondary to damage rather than a primary event, their differential induction in either area of the CNS does not necessarily mean that WM and GM lesions are driven by different pathogenic pathways. The difference does suggest, however, that GM areas in an MS-affected CNS benefit much less from the protective functions of small HSPs than WM areas. This, in turn, may suggest that therapeutic intervention involving augmentation of the functions of small HSPs may be particularly helpful to MS patients presenting with cortical damage.

## Additional file

Additional file 1:
**Figure S1.** Representative images of white matter tissues from the same non-neurological control and MS cases as used to examine expression of other small HSPs. Double immunohistochemistry staining for HLA-DR (pink) and HSPB5 (brown). Control white matter (A) and NAWM (B), NAWM surrounding a preactive lesion (C), preactive lesion (D), active lesion (E), chronic active lesion (F- rim, G - centre) and inactive lesions (H) from MS cases. Scale bar = 50μm (PDF 390 kb)

## Abbreviations

AF: alkaline phosphatase
CNS: central nervous system
CON: control
DAB: 3,3’-diaminobenzidine
DAPI: 4',6-diamidino-2-phenylindole
E: efficiency
EF1α: elongation factor 1α
GFAP: glial fibrillary acidic protein
GM: grey matter
HLA: human leukocyte antigen
HRP: horse radish peroxidase
HSPs: heat-shock proteins
LC: leukocortical lesions
NAGM: normal appearing grey matter
NAWM: normal appearing white matter
MS: multiple sclerosis
PBS: phosphate buffered saline
PCR: polymerase chain reaction
PLP: proteolipid protein
RT: room temperature
SEM: standard error of the mean
TBS: tris buffered saline
TLR: toll-like receptor
WM: white matter
