# Supplementary material for: Small heat shock proteins are induced during multiple sclerosis lesion development in white but not grey matter
Source: Acta Neuropathol Commun. 2015 Dec 22;3:87. doi: 10.1186/s40478-015-0267-2 (PMC4688967; doi:10.1186/s40478-015-0267-2)
Supplement: Additional file 1: — Figure S1. Representative images of white matter tissues from the same non-neurological control and MS cases as used to examine expression of other small HSPs. Double immunohistochemistry staining for HLA-DR (pink) and HSPB5 (brown). Control white matter (A) and NAWM (B), NAWM surrounding a preactive lesion (C), preactive lesion (D), active lesion (E), chronic active lesion (F- rim, G - centre) and inactive lesions (H) from MS cases. Scale bar = 50μm (PDF 390 kb) [file 40478_2015_267_MOESM1_ESM.pdf]

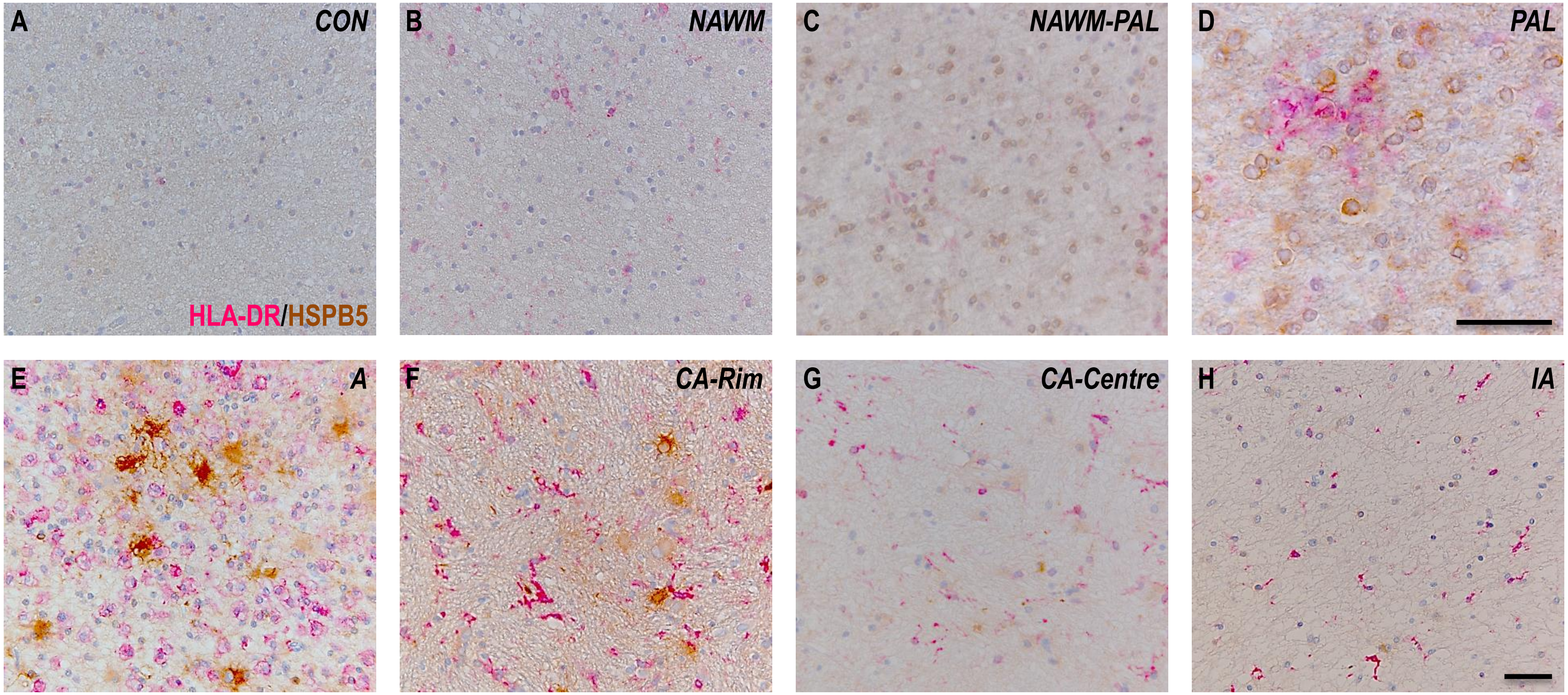

Supplementary Figure 1. Representative images of white matter tissues from the same non-neurological control and MS cases as used to examine expression of other small HSPs. Double immunohistochemistry staining for HLA-DR (pink) and HSPB5 (brown). Control white matter (**A**) and NAWM (**B**), NAWM surrounding a preactive lesion (**C**), preactive lesion (**D**), active lesion (**E**), chronic active lesion (**F**- rim, **G** - centre) and inactive lesions (**H**) from MS cases. Scale bar = 50µm
